# Computational modeling suggests dimerization of equine infectious anemia virus Rev is required for RNA binding

**DOI:** 10.1186/s12977-014-0115-7

**Published:** 2014-12-23

**Authors:** Chijioke N Umunnakwe, Hyelee Loyd, Kinsey Cornick, Jerald R Chavez, Drena Dobbs, Susan Carpenter

**Affiliations:** Department of Animal Science, Iowa State University, Ames, IA 50011 USA; Department of Genetics, Developmental and Cell Biology, Iowa State University, Ames, IA 50011 USA; Program in Bioinformatics and Computational Biology, Iowa State University, Ames, IA 50011 USA; Department of Biochemistry, Biophysics and Molecular Biology, Iowa State University, Ames, IA 50011 USA

**Keywords:** Rev, Bipartite RNA binding domain, EIAV, Lentivirus, Dimerization, Coiled-coil motif, Arginine-rich motif

## Abstract

**Background:**

The lentiviral Rev protein mediates nuclear export of intron-containing viral RNAs that encode structural proteins or serve as the viral genome. Following translation, HIV-1 Rev localizes to the nucleus and binds its cognate sequence, termed the Rev-responsive element (RRE), in incompletely spliced viral RNA. Rev subsequently multimerizes along the viral RNA and associates with the cellular Crm1 export machinery to translocate the RNA-protein complex to the cytoplasm. Equine infectious anemia virus (EIAV) Rev is functionally homologous to HIV-1 Rev, but shares very little sequence similarity and differs in domain organization. EIAV Rev also contains a bipartite RNA binding domain comprising two short arginine-rich motifs (designated ARM-1 and ARM-2) spaced 79 residues apart in the amino acid sequence. To gain insight into the topology of the bipartite RNA binding domain, a computational approach was used to model the tertiary structure of EIAV Rev.

**Results:**

The tertiary structure of EIAV Rev was modeled using several protein structure prediction and model quality assessment servers. Two types of structures were predicted: an elongated structure with an extended central alpha helix, and a globular structure with a central bundle of helices. Assessment of models on the basis of biophysical properties indicated they were of average quality. In almost all models, ARM-1 and ARM-2 were spatially separated by >15 Å, suggesting that they do not form a single RNA binding interface on the monomer. A highly conserved canonical coiled-coil motif was identified in the central region of EIAV Rev, suggesting that an RNA binding interface could be formed through dimerization of Rev and juxtaposition of ARM-1 and ARM-2. In support of this, purified Rev protein migrated as a dimer in Blue native gels, and mutation of a residue predicted to form a key coiled-coil contact disrupted dimerization and abrogated RNA binding. In contrast, mutation of residues outside the predicted coiled-coil interface had no effect on dimerization or RNA binding.

**Conclusions:**

Our results suggest that EIAV Rev binding to the RRE requires dimerization via a coiled-coil motif to juxtapose two RNA binding motifs, ARM-1 and ARM-2.

**Electronic supplementary material:**

The online version of this article (doi:10.1186/s12977-014-0115-7) contains supplementary material, which is available to authorized users.

## Background

The Rev protein of lentiviruses mediates nuclear export of singly spliced and unspliced viral RNA transcripts. HIV-1 Rev binds its target RNA, the Rev responsive element (RRE), as a monomer, then multimerizes along the RRE RNA before being shuttled to the cytoplasm through association with the Crm1 host export factor. Several well-characterized motifs mediate known functions of HIV-1 Rev: a nuclear localization signal (NLS), which overlaps an RNA-binding arginine-rich motif (ARM); a nuclear export signal; and a pair of oligomerization domains that flank the ARM (reviewed in [1]).

RNA recognition by HIV-1 Rev is mediated by a 17-residue long ARM that adopts an alpha-helical conformation and initially docks into the major groove of a highly structured region in the HIV-1 RRE, termed stem loop IIB (SLIIB) [2-6]. Biochemical and biophysical studies have revealed that HIV-1 Rev oligomerizes [7,8] and that monomeric, dimeric, and higher-order oligomeric forms associate with the RRE [9-12]. These and subsequent studies [13] have shown that HIV-1 Rev binding to the RRE is a stepwise process: initial binding of Rev to SLIIB acts as a nucleation event that drives further oligomerization of additional copies of Rev along the RRE (reviewed in [14]). Although, monomeric HIV-1 Rev has been shown to bind the RRE in gel shift, filter binding, and single molecule fluorescence spectroscopy assays [9,12,15,16], studies of the tertiary structure of HIV-1 Rev and the RRE suggest that the “fundamental building block” for RRE binding is a Rev dimer [15,17]. Both dimeric (head-to-head) contacts and higher-order oligomeric (tail-to-tail) intermolecular contacts are critical for Rev-mediated RNA export [12,18-22].

NMR studies of HIV-1 Rev revealed that the C-terminal half of the protein is intrinsically disordered [13]; however, crystal structures of the N-terminal half of HIV-1 Rev, including the ARM and oligomerization motifs, have provided valuable insights into the structural basis of RNA binding and multimerization [13,15,23]. In the crystal structures, the N-terminal half of the Rev monomer adopts a helix-loop-helix structure with hydrophobic patches on opposite surfaces. Hydrophobic patches on one surface contain residues that drive dimerization, whereas hydrophobic patches on the opposite surface contain residues that mediate oligomerization (reviewed in [22,24]). Dimerization of HIV-1 Rev orients monomers in a ‘V’ shape with an angle of 120-140° and a distance of ~55 Å between the distal ends [15,23]. Recent SAXS analysis [17] indicates that the HIV-1 RRE adopts an unusual topology resembling the letter ‘A’, with the ‘legs’ forming Rev binding tracks. The ‘legs’ are spaced ~55 Å apart and appear to match the distance between the ARMs in an HIV-1 Rev dimer [15,17,23]. Although, Rev monomers can bind the HIV-1 RRE, it is believed that the specific structural arrangement of Rev dimers combined with the complementary topology of the RRE dictates Rev-RRE binding specificity and aids recognition of cognate RNA substrate from among an abundant pool of host RNAs [13,15,17,23].

Equine infectious anemia virus (EIAV) Rev is functionally homologous to HIV-1 Rev, but shares very little sequence similarity and differs in domain organization (reviewed in [25]). We previously identified a bipartite RNA binding domain in EIAV Rev that contains two short arginine-rich motifs (designated ARM-1 and ARM-2) spaced 79 amino acids apart in the primary sequence [26]. ARM-1 is located in the central region of the protein while ARM-2 resides at the C-terminus and also functions as an NLS. It is possible that ARM-1 and ARM-2 are in close proximity in the Rev monomer, forming a single RNA-binding interface. Alternately, ARM-1 and ARM-2 could each bind different sites on the RRE RNA. RNA footprinting and chemical modification experiments have shown that the RRE target of EIAV Rev contains two Rev binding regions (designated RBR-1 and RBR-2) that undergo conformational changes in the presence of EIAV Rev [27]. RBR-1 encompasses the minimal RRE sequence, which overlaps a characterized exonic splicing enhancer [28-30], while RBR-2 is necessary for high-affinity Rev binding *in vitro* [27].

Insight as to how the bipartite RNA binding domain interacts with the RRE target requires knowledge of the tertiary structure of EIAV Rev and relative positioning of ARM-1 and ARM-2 in the folded protein. Obtaining high-resolution structures of Rev proteins has proven very challenging due to the tendency of Rev to spontaneously aggregate into insoluble filaments in solution [7-9]. Computational modeling of the EIAV Rev structure has been challenging, also, because the amino acid sequence similarity between HIV-1 Rev and EIAV Rev is almost undetectable [31]. Thus, it is not possible to simply use the available experimental structures of HIV-1 Rev as templates for homology modeling of non-primate Rev proteins. Recent progress in *ab initio* and threading methods for structure prediction, however, has provided a viable platform for modeling structures of proteins that have proven difficult to characterize experimentally [32-40].

The first proposed structural model for EIAV Rev suggested that ARM-1 and ARM-2 are juxtaposed to form a single RNA binding interface on the monomer structure [31]. In the present study, newer and more accurate structural modeling approaches were employed to predict the topology and relative orientation of ARM-1 and ARM-2 within the overall structure of EIAV Rev. Our results suggest that ARM-1 and ARM-2 do not form a single RNA binding interface within a single Rev monomer. Instead, our computational analyses, supported by experimental data, suggest that dimerization of Rev is a prerequisite for RNA binding. Thus, dimerization of EIAV Rev may be required to juxtapose ARMs from two Rev monomers so that they form a single functional RNA binding domain that recognizes the EIAV RRE.

## Results

### Generation of Rev structural models

**Figure 1:**
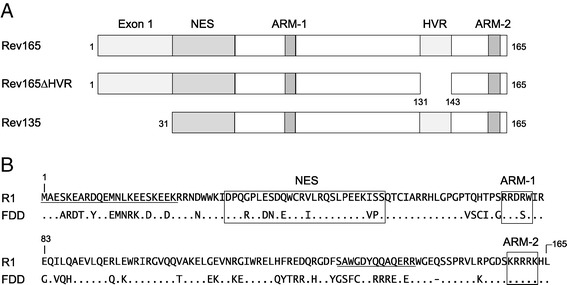
**Rev sequences used for computational prediction of tertiary structures. A**. Schematic representations of the reference EIAV R1 Rev165 and R1 deletion mutants, Rev∆HVR and Rev135. The residue numbers indicate N and C termini and boundaries of deleted regions. The locations of functional motifs are indicated: NES: nuclear export signal, ARM: arginine-rich motif, HVR: hypervariable region. **B**. Alignment of Rev165 and RevFDD, derived from the fetal donkey dermal cell-adapted Chinese strain EIAV_FDD-10_ [44]. Sequences shown to be non-essential for nuclear export activity (exon1 and HVR [26]) are underlined, while characterized functional motifs (NES, ARM-1, and ARM-2) are boxed.

A computational approach was employed to model the tertiary structure of EIAV Rev and obtain insight into the topology of the bipartite RNA binding domain. Because EIAV Rev is highly variable in sequence and contains non-essential regions (reviewed in [41]), our analyses included deletion mutants as well as a divergent Rev variant (Figure 1). Rev165 is the full-length sequence of the R1 variant derived from the EIAV_Wyo2078_ field isolate [42]; Rev135 contains an N-terminal deletion of R1 encompassing all of exon 1, while Rev∆HVR contains a 13-amino-acid deletion in the hypervariable region, located in the C-terminal half of the protein [26,43]. Rev135 and Rev∆HVR are functionally equivalent to Rev165 in *in vitro* assays of nuclear export activity [26]. RevFDD is from the fetal donkey dermal cell-adapted Chinese isolate EIAV_FDD-10_ [44]. FDD Rev and Rev165 differ at 54 positions across the length of the protein (Figure 1B), demonstrating marked variation in primary amino acid sequence.

A total of 235 computational models were generated using several state-of-the-art protein structure prediction servers that implement different algorithms (Table 1). These include the QUARK server, which implements an *ab initio* algorithm [36,37]; the ITASSER and LOMETS servers, which implement threading-based algorithms [34,45]; and the PROTINFO server, which implements an homology modeling algorithm [46]. Examination of the predicted models revealed significant variation in their overall shapes (Table 1): 52% of the Rev165 models had an elongated topology, 34% had a globular topology, and 14% were either unfolded and/or truncated. Models generated for Rev135, Rev∆HVR, and RevFDD also showed elongated and globular topologies, in proportions comparable to those for Rev165. The topology of models obtained depended, in part, on the method of structure prediction: the QUARK *ab initio* server predominately yielded elongated topologies, whereas homology servers generated exclusively globular topologies. A mixture of elongated and globular topologies was generated by the ITASSER and LOMETS threading servers.

**Table 1:** **Computational prediction of Rev structural models**

**EIAV Rev sequence**	**Computational method**	**Server**	**Topology of models**			**Total**
			**Elongated** ^**a**^	**Globular** ^**b**^	**Unstructured** ^**c**^	
Rev165	*Ab initio*	QUARK	20	0	0	20
	Threading	ITASSER	16	14	0	30
		LOMETS	6	4	10	20
	Homology	PROTINFO	0	6	2	8
Rev135	*Ab initio*	QUARK	28	0	0	28
	Threading	ITASSER	7	17	1	25
		LOMETS	3	9	8	20
	Homology	PROTINFO	0	1	3	4
Rev∆HVR	*Ab initio*	QUARK	19	1	0	20
	Threading	ITASSER	4	16	0	20
		LOMETS	3	8	9	20
RevFDD	*Ab initio*	QUARK	10	0	0	10
	Threading	ITASSER	5	5	0	10
Totals			121	81	33	235

^a^Elongated: models with an unbroken extended alpha helix in the central region.

^b^Globular: models with kinks in the central region.

^c^Unstructured: models with an unfolded topology or missing C-terminal residues encompassing ARM-2.

### Assessment and structural features of Rev models

**Figure 2:**
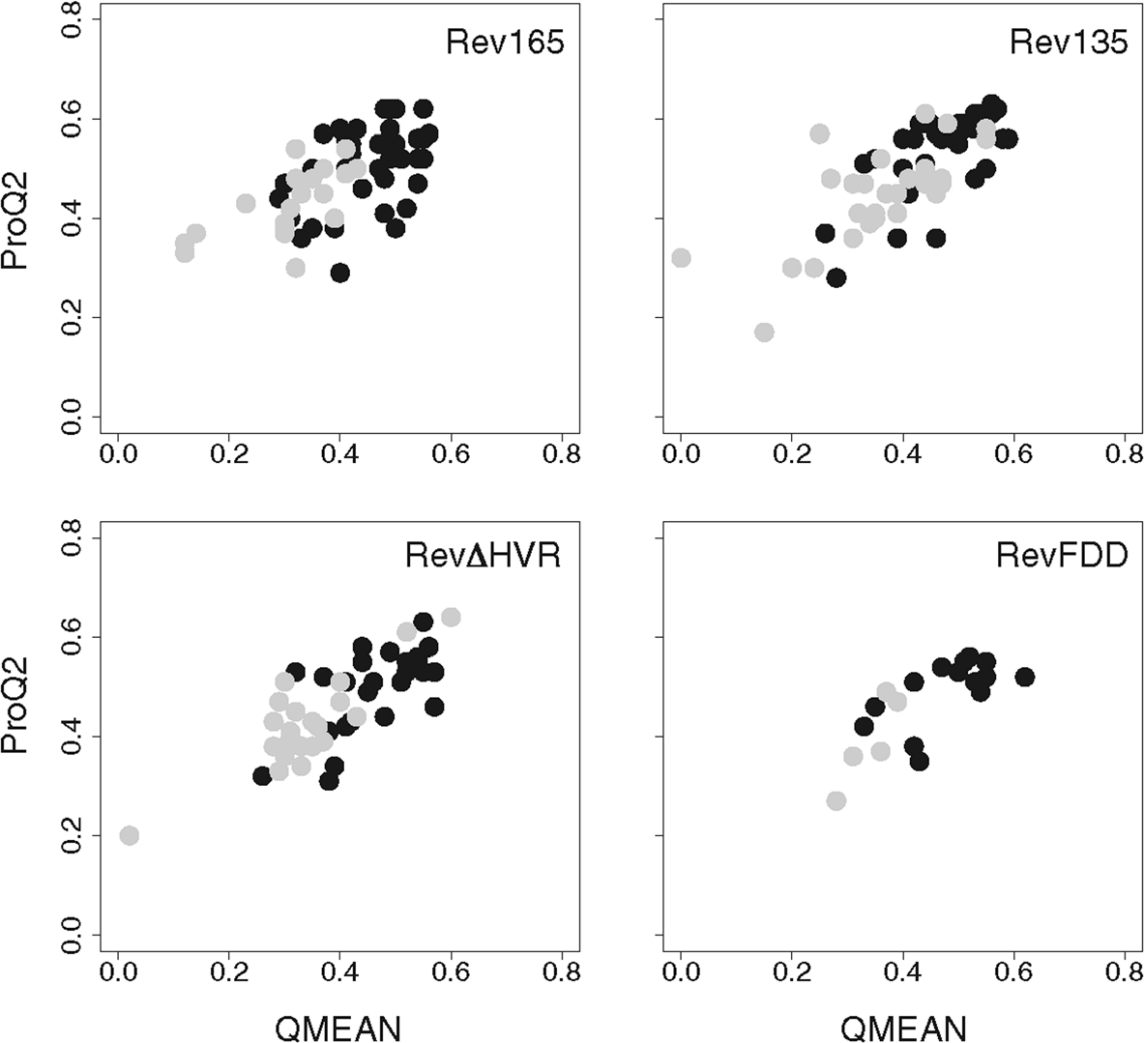
**Comparison of quality assessment scores for elongated versus globular Rev structural models.** Quality assessment scores for all Rev models were obtained using QMEAN [47,48] and ProQ2 [49]. Each graph shows the distribution of model quality scores for elongated models (black circles) versus globular models (grey circles) for the indicated Rev protein sequence. Quality scores range from 0 (worst) to 1 (best) for both QMEAN and ProQ2. The score distributions for elongated and globular models displayed considerable overlap, although elongated models tended to have higher scores.

The quality of generated models was assessed using the QMEAN and ProQ2 model quality assessment programs [47-49] (see Additional file 1). These programs evaluate the physicochemical and structural features of a given model by comparison with those of known experimental structures. Both programs generate a score in the range 0–1, with 1 signifying the highest possible quality score. The calculated QMEAN score for each model was plotted against its corresponding ProQ2 score and the distribution of model quality scores for each Rev sequence analyzed is shown in Figure 2. The majority of models have QMEAN and ProQ2 scores ~0.5, indicating most are of average quality. Although the elongated models generally scored higher than globular models, the overlap in quality scores precluded selection of a single preferred tertiary topology.

**Figure 3:**
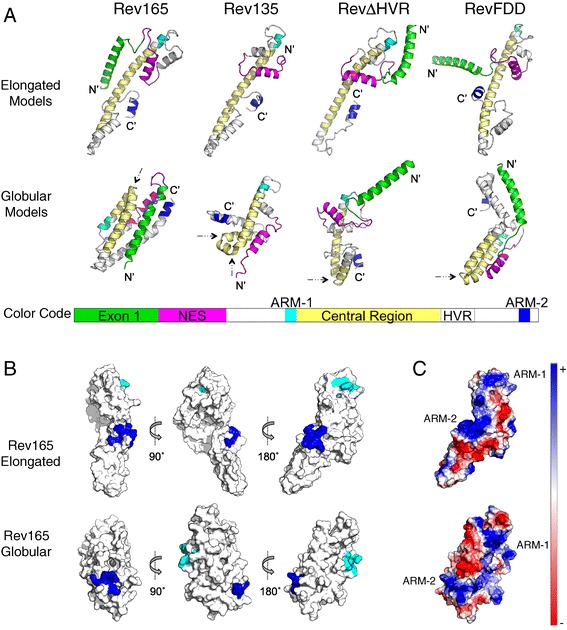
**Structural features of Rev models. A**. Cartoon representations of the top-scoring elongated and globular models for each of the four EIAV Rev sequences analyzed. The elongated models share a similar overall fold, with the defining structural feature being an extended alpha helix in the central region (colored yellow). The globular models are defined by a ‘hinged’ fold, wherein the central region is disrupted by a kink, indicated by black arrows. The color code used for visualizing the models is shown below in the context of the domain structure of Rev165. **B**. Relative positioning of ARM-1 and ARM-2 in top scoring elongated and globular Rev165 models, showing three different rotational angles. In all three angles, ARM-1 and ARM-2 are well separated in the tertiary structure, and are on opposite faces. **C**. Electrostatic surface representation corresponding to the right-most rotational view of Rev165 shown in Figure 3B. Negative charges on the protein surface are colored red and positive charges are colored blue. The patch of positive charge bridging ARM-1 and ARM-2 consists of residues from exon1, which can be deleted with no effect on Rev function *in vivo*.

Key structural features in EIAV Rev elongated and globular models were identified by visual inspection using PyMol software [50]. Figure 3A shows representative examples of the top ranking elongated and globular models for Rev165, Rev135, Rev∆HVR, and RevFDD. A distinguishing structural feature common to virtually all elongated models was an extended alpha helix in the central region of the protein. In globular models, this central helix was disrupted by a kink (Figure 3A, black arrows), resulting in a compact bundle of helices. In all models, ARM-1, ARM-2, and exon1 adopted alpha-helical conformations (Figure 3A). The NES formed a short alpha helix flanked on both sides by flexible loops, which usually formed a helix-turn-helix motif with the adjacent helix. ARM-1 was always positioned at the N-terminus of the central region. The other functional motifs were separated by flexible regions, and the positioning of these motifs relative to the central region was the major difference among the various models in both elongated and globular structures. The greatest variability was observed in the position of exon1 (Figure 3A), which is rich in solvent-exposed, hydrophilic residues.

### Relative positioning of the bipartite RNA binding domain in Rev models

PyMol software [50] was used to inspect the relative positioning of ARM-1 and ARM-2 on the surface of the predicted structures. The distance separating the two closest atoms in ARM-1 and ARM-2 was calculated for each model (Additional file 1). In 204 of 235 generated models, ARM-1 and ARM-2 were separated by ≥15 Å on the monomer surface. In addition, ARM-1 and ARM-2 were positioned on opposite faces of the monomer in many of the top structures (Figure 3B). Although, electrostatic views show that the two ARMs could be bridged by a continuous stretch of positive charge in some models (Figure 3C), the bridging region consisted of positively charged residues from exon1, which can be deleted with no loss of Rev activity *in vitro* [26]. These results strongly suggest that ARM-1 and ARM-2 do not form a single RNA binding interface in the Rev monomer.

### A coiled-coil motif in EIAV Rev may promote dimerization

**Figure 4:**
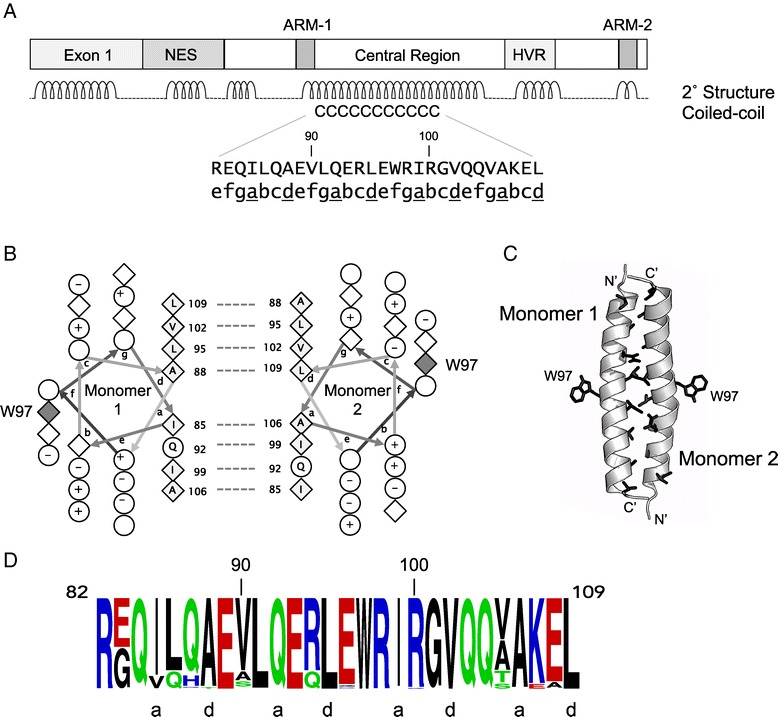
**An identified coiled-coil motif in EIAV Rev is predicted to mediate dimerization. A**. Domain organization and secondary structure prediction for EIAV Rev165, showing the location of the predicted coiled-coil motif in the central region (residues 82–109). The coiled-coil motif is within a predicted extended alpha helix. Amino acid residues of the coiled-coil motif heptad repeats are shown in upper case. The register (*abcdefg*) of each residue is shown in lower case, and ‘*a*’ and ‘*d*’ registers, which are critical for interfacial interactions [56,57], are underlined. **B**: Helical wheel representation [58] of predicted intermolecular interactions mediated by ‘*a*’ and ‘*d*’ registers of the coiled-coil motif. Diamonds represent hydrophobic residues; + denotes positively charged polar residues, − denotes negatively charged polar residues; open circles represent uncharged polar residues. Dashed lines connect pairs of hydrophobic residues predicted to make interfacial contacts. Filled diamonds are Trp residues that could participate in oligomerization. **C**. Cartoon illustrating head-to-tail dimeric structure generated by ClusPro docking of two EIAV Rev fragments corresponding to the coiled-coil motif. Side chains of ‘*a*’ and ‘*d*’ residues predicted to make interhelical contacts in **(B)** are shown as black sticks. Note that Trp residues (W97) that could potentiate oligomerization are exposed on opposite faces of the docked structure. **D**. Sequence conservation in the coiled-coil motif. The sequence logo of residues 82–109 of EIAV Rev was generated from a multiple sequence alignment of 200 EIAV Rev isolates from US, Ireland, and China using WebLogo [59]. Stacks of letters at each position indicate the relative frequency of an amino acid in the multiple sequence alignment. Six of the 8 residues in the ‘*a*’ and ‘*d*’ positions are invariant while the Ile in the first ‘*a*’ position accommodates only Val, a closely related hydrophobic residue.

Given that ARM-1 and ARM-2 are not predicted to form a single RNA binding interface on the Rev monomer, two scenarios for RNA binding are possible: i) ARM-1 and ARM-2 form two distinct RNA binding interfaces, or ii) dimerization of Rev juxtaposes ARM-1 from one monomer with ARM-2 from a second monomer to form a single RNA binding interface. The latter scenario predicts that EIAV Rev dimerizes, and that dimerization is essential for RNA binding. Therefore, the primary sequence of EIAV Rev was computationally analyzed for oligomerization motifs [51-54]. Results identified a canonical coiled-coil motif, spanning residues 82–109, within an extended alpha helix predicted in the central region of Rev (Figure 4A). The predicted coiled-coil motif displayed characteristics typical for an oligomerization domain [51,52,55,56], with hydrophobic residues predominantly occupying ‘*a*’ and ‘*d*’ registers of the coiled-coil and charged residues preferentially occurring in ‘*e*’ and ‘*g*’ registers.

A helical wheel projection of the predicted coiled-coil motif (Figure 4B) shows that the ‘*a*’ and ‘*d*’ residues (Leu, Ile, Val, Ala) constitute the hydrophobic face of an amphipathic helix and are well positioned to mediate dimerization. Additionally, a bulky Trp residue is predicted to reside on the opposite side of the interhelical interface. Docking of predicted coiled-coil structures using the ClusPro server [60-63] resulted in formation of a head-to-tail dimer, with residues in the ‘*a*’ and ‘*d*’ registers forming an interhelical interface and the bulky Trp residue segregating to the opposite face, in a position where it could mediate further oligomerization (Figure 4C). Although both head-to-head and head-to-tail orientations were obtained by docking, the head-to-tail orientation resulted in a larger number of contacts between hydrophobic ‘*a*’ and ‘*d*’ residues and a more energetically favorable dimer structure. Fewer interactions between ‘*a*’ and ‘*d*’ residues of the coiled-coil were observed when docking full-length elongated structures, in either the head-to-head or head-to-tail orientation (not shown).

### The coiled-coil motif is highly conserved among EIAV Rev variants

There is high degree of genetic variation in EIAV Rev sequences (reviewed in [41]), and it was of interest to examine conservation of residues in the predicted coiled-coil motif. Accordingly, 200 distinct Rev amino acid sequences encompassing phylogenetically diverse isolates were retrieved from GenBank (Additional file 2), aligned, and analyzed using the WebLogo server [59]. These analyses revealed that a large number of residues in the predicted coiled-coil region are, in fact, invariant (Figure 4D). More importantly, residues in the ‘*a*’ and ‘*d*’ positions are either completely conserved, or were substituted only with similarly hydrophobic residues. The high degree of conservation suggests that the predicted coiled-coil motif contributes an essential function in Rev activity. In support of this, mutation of hydrophobic residues located in the predicted interhelical interface (L95D, L109D) abrogated Rev activity, whereas mutation of hydrophobic residues that lie outside the predicted interface (e.g., V112D) retained wild-type Rev activity [31,43].

### Dimerization is required for RNA binding in EIAV Rev

**Figure 5:**
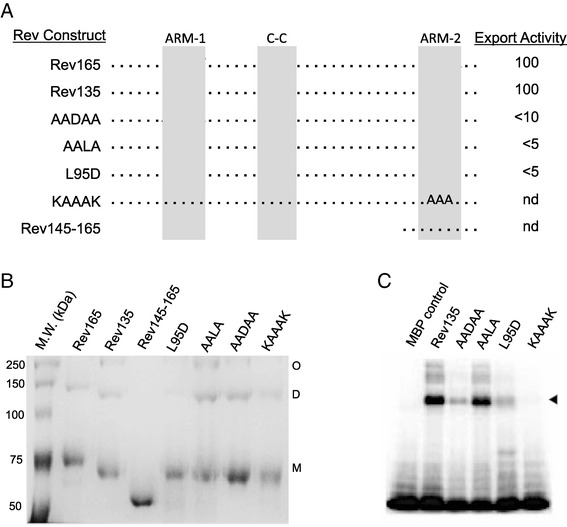
**Specific residues within the predicted coiled-coil motif are required for dimerization and RNA binding.** Representation of MBP-Rev constructs evaluated for dimerization and RNA binding. Rev165 is the reference construct used for all comparisons; Rev135 contains a 30 aa deletion of the non-essential exon1 region, and Rev145-165 contains only the 21 C-terminal amino acid residues of Rev. Indicated mutations in ARM-1, ARM-2 and the coiled-coil (C-C) motif were introduced into Rev135. Nuclear export activity of Rev cDNAs containing each mutation measured in a previous study is indicated [26], nd: not determined. B. Oligomeric forms of purified MBP-Rev proteins. The purified MBP-Rev proteins were analyzed by Coomassie-stained Blue-native PAGE in the presence of 0.2% SDS. The L95D mutation in the predicted interhelical interface abolishes dimerization whereas mutation of residues flanking L95 (AALA) does not affect dimerization. Mutation of ARM-1 (AADAA) and or ARM-2 (KAAAK) does not affect dimerization. C. RNA-binding activity as measured by UV cross-linking and SDS-PAGE (reproduced from [26]). The L95D mutation in the predicted coiled-coil interhelical interface causes a dramatic decrease in RNA binding, whereas mutation of residues flanking L95 (AALA mutant) does not affect RNA binding. Marked reduction of RNA binding activity was also observed in ARM-1 and ARM-2 mutants (AADAA and KAAAK, respectively).

Coiled-coil motifs generally mediate intermolecular interactions and, less frequently, intramolecular interactions [56-58]. In the context of our predicted EIAV Rev structures, intermolecular coiled-coil interactions would be favored by elongated structures whereas intramolecular coiled-coil interactions would occur in globular structures. In the elongated structures, interactions between coiled-coil motifs in two different Rev monomers could juxtapose ARM-1 and ARM-2 to form a single RNA binding interface in a dimeric structure. In the globular models, intramolecular interactions between the two smaller helices in the center of the monomer could be important for structural stability [31]. To test the hypothesis that the Rev coiled-coil motif mediates dimerization, purified MPB-Rev fusion proteins containing mutations in the predicted coiled-coil interface were analyzed by Blue native PAGE (Figure 5). Because Rev aggregates readily in solution, samples were resuspended in Blue native PAGE loading buffer supplemented with 0.2% SDS. MBP-Rev165 and MBP-Rev135 samples migrated as monomeric, dimeric and higher oligomeric forms (Figure 5B). In contrast, only the monomeric form was present in samples of MBP-Rev145-165, which contains only the C-terminal 21 amino acids of Rev. Aspartic acid substitution of Leu 95, which is predicted to be critical for mediating intermolecular coiled-coil interactions, resulted in loss of dimerization, whereas alanine substitutions of non-interfacial residues in the coiled-coil motif (ERLE to AALA) had no effect on dimerization (Figure 5B). These data support the hypothesis that the EIAV Rev coiled-coil motif mediates intermolecular interactions between Rev monomers, resulting in formation of dimers.

To explore the importance of dimerization for RNA binding, we re-examined previous studies that mapped determinants of EIAV Rev required for RNA binding [26]. In UV-crosslinking experiments, the L95D mutation, which abolishes dimerization, resulted in markedly reduced RNA binding activity, whereas the AALA mutant in which dimerization is not affected retained wild-type binding activity (compare Figures 5B and C). Thus, loss of Rev dimerization is correlated with loss of RNA binding activity. Furthermore, mutations within ARM-1 and ARM-2 that disrupted RNA binding did not affect dimerization (Figure 5B,C), indicating that dimerization and RNA binding are distinct and separable functions of Rev. Taken together, these results indicate that a coiled-coil motif mediates dimerization of EIAV Rev, and that dimerization is a prerequisite for Rev binding to the RRE.

## Discussion

The Rev protein of EIAV contains a bipartite RNA binding domain, containing two arginine-rich motifs, designated ARM-1 and ARM-2, which are separated by 79 residues in the amino acid sequence. In this study, computational models were generated and evaluated in an effort to determine the relative positioning of ARM-1 and ARM-2 on the tertiary structure of EIAV Rev. Two overall topologies for the Rev monomer were predicted: an elongated structure with an extended central alpha helix, and a globular structure with a kink in the central helix, resulting in a bundle of helices. In 204 of 235 generated models, ARM-1 and ARM-2 were well separated on the tertiary structure, strongly suggesting that a single RNA binding interface is not formed on the Rev monomer. A highly conserved coiled-coil motif was identified in the central region of EIAV Rev and was found to mediate dimerization of Rev monomers *in vitro*. Mutation of residues predicted to form key intermolecular coiled-coil contacts abolished dimerization and also disrupted RNA binding. In contrast, mutation of residues predicted to lie outside the coiled-coil interface had no effect on dimerization or RNA binding activity. Taken together, our results suggest that the EIAV Rev monomer adopts an elongated structure that dimerizes through intermolecular interactions mediated by a highly conserved coiled-coil motif in the central region of the protein. Dimerization is predicted to juxtapose ARM-1 from one monomer with ARM-2 from a second monomer to form a single RNA binding interface.

The central region of Rev is known to be sensitive to mutation [26], but a specific role for this region in the Rev nuclear export pathway has not been identified. The presence of a highly conserved coiled-coil motif in the central region suggests that it is required for intermolecular and/or intramolecular interactions essential for Rev activity. In elongated structural models, the coiled-coil is positioned to meditate intermolecular interactions required for dimerization and RNA binding; in the globular models, the coiled-coil motif would mediate intramolecular interactions that contribute to protein stability. Our data are most consistent with an elongated topology wherein the coiled-coil motif mediates formation of a Rev dimer. In this scenario, coiled-coil intermolecular interactions that stabilize the EIAV Rev dimer are maximized in an antiparallel orientation, suggesting that EIAV Rev binds RNA as a head-to-tail dimer. In support of this model, a series of *trans*-complementation experiments reported by Harris *et al*., [64] showed that co-transfection of ARM-1 and ARM-2 mutants, each deficient for RNA export, restored Rev activity. In contrast, *trans*-complementation was abolished by mutation of residues that correspond to key contacts in the coiled-coil interface. In total, the computational models and experiments reported here, combined with previous experimental results, indicate that a coiled-coil motif in the central region of EIAV Rev mediates dimerization of Rev, which in turn, plays an essential role in RNA binding and Rev activity.

The predicted overall fold of EIAV Rev reported here shows both similarities and differences compared with the crystal structure of the HIV-1 Rev monomer [15,23]. In both Rev proteins, the ARM motifs adopt an alpha-helical conformation. Oligomerization domains are found in both proteins and play an essential role in Rev function. The oligomerization domain of EIAV Rev contains the strong signature of a coiled-coil motif, which is required for dimerization and binding to the RRE. A corresponding canonical coiled-coil motif is not found in HIV-1 Rev. Instead, hydrophobic residues flanking the ARM mediate oligomerization of Rev on the RRE [13,15,16]. One difference between the two lentiviral Rev proteins is that dimerization is required for RNA binding of EIAV, but not HIV-1, Rev *in vitro*. In both cases, however, dimerization may be the biologically relevant configuration that determines RNA-binding specificity and formation of a functional nuclear export complex *in vivo*.

Our study highlights the value of employing computational methods to gain insight into structure-function relationships of Rev proteins, which have proven extremely difficult to characterize experimentally. In particular, recent advances in *ab initio* and threading based modeling has resulted in increased power and accuracy in predicting protein structure. *Ab initio* methods have the advantage of not requiring a structural template that shares sequence homology to that of the protein of interest; current *ab initio* methods can reliably predict tertiary structures of proteins ≤ 200 amino acids in length [36-38,40]. Model quality assessment has also improved significantly in recent years and provides a quantitative measure of confidence in the quality of predicted protein structures [65,66]. Due to the low level of sequence identity between EIAV and HIV-1 Rev and the lack of other homologous templates, the “average” scores of our predicted models were not unexpected. The quality score of a given model depends in part, on whether the overall fold of the model is consistent with predictions of secondary structure generated by independent methods [47-49]; therefore, models of average quality can yield useful information on general topology and spatial features of a protein. The elongated topology is most consistent with secondary structure predictions, in which the central region of EIAV Rev adopts an extended alpha helical conformation (Figure 4A). This explains, in part, why the elongated models generally scored higher than globular models, especially those generated by *ab initio* servers. Although we were unable to select a single topology based on computational predictions alone, both the globular and elongated models indicate that ARM-1 and ARM-2 do not form a single RNA binding interface, a finding that motivated the search for an oligomerization motif in EIAV Rev. It will be of interest to determine whether coiled-coil motifs are found in other retroviral Rev or Rev-like proteins where they may contribute to oligomerization and nuclear export activity.

## Conclusion

This study provides computational and experimental data indicating that dimerization of EIAV Rev is required for RNA binding. Our results suggest dimerization is mediated by a coiled-coil motif in the central region of Rev. This work illustrates that computational modeling, combined with a molecular genetics approach, can be a valuable tool for interrogating the tertiary structure of Rev proteins and generating testable hypotheses regarding the mechanisms by which lentiviral Rev proteins recognize and bind their cognate RNA targets.

## Methods

### Generation of EIAV Rev structural models

#### Sequences

EIAV Rev R1 [GenBank:AAG53100] was used as the reference amino acid sequence for generating full-length EIAV Rev165 structural models. R1 was isolated from a pony experimentally infected with EIAV_Wyo2078*,*_ a highly virulent strain of EIAV [42]. Additional Rev sequence variants included: R1 Rev135, which lacks the first 30 amino acids encoded by exon1; R1 Rev∆HVR, in which the hypervariable region (residues 131–143) is deleted [26,43]; and RevFDD [44], the full-length Rev sequence from the Chinese isolate EIAV_FDD-10_ [GenBank:ADK35837].

#### Servers

The QUARK, ITASSER, LOMETS, and PROTINFO protein structure prediction servers were used for automated modeling of Rev and are described in [32,34,36,37,45,46]. Default settings were used for the QUARK, ITASSER, and LOMETS servers. The “*generate comparative models*” option was used for PROTINFO. For the ITASSER server, in addition to default settings, Rev was modeled using an HIV Rev crystal structure (PDB:3lph) [15] as the specified template with two different parameterized settings: i) the “*specify template without an alignment*” mode; and ii) the “*specify template with alignment*” mode. Pairwise alignment of R1 and HIV-1 Rev 3lph:A amino acid sequences was generated with the T-Coffee webserver [67]. All models were manually inspected and models with an unfolded topology or those missing C-terminal residues encompassing ARM-2 were excluded from further analysis.

### Quality assessment of EIAV Rev structural models

The QMEAN [47,48] and ProQ2 [49] servers were used to evaluate models for consistency with known protein structural features. These are among the top performing model quality assessment servers, routinely outperforming other assessment programs in recent CASP competitions [48,49,65,66]. To discriminate between high and low quality models, QMEAN uses a composite scoring function based on four geometrical features: i) local geometry, ii) long-range interactions, iii) all-atom potential, and iv) solvation energy of residues [47,48]. The output score ranges from 0 to 1, where 1 is the highest score. The mean scores of high, medium and low quality models are 0.68, 0.58, and 0.40, respectively [47,48]. ProQ2 predicts both local and global “correctness” of models using a support vector machine algorithm that considers the following features of a given model: i) atom-atom and residue-residue contacts, ii) solvent accessibility, iii) predicted secondary structure, iv) predicted surface area exposure, and v) evolutionary information [49]. The quality of a model predicted by ProQ2 is consistent with predictions by QMEAN [49]. All models generated in this study were evaluated with both servers, using default parameters.

### Alignment of EIAV Rev protein sequences

Pairwise protein alignments were performed with the T-Coffee webserver [67], using the default settings of the T-Coffee mode. Multiple sequence alignments were performed with MacVector software, using the Gonnet substitution matrix with default settings [68,69].

### Prediction of coiled-coil motifs

Coiled-coil motif prediction was performed using the COILS [51,52], PAIRCOIL [53], and CCHMMPROF [54] servers. For the COILS server, the following parameters were used: a 28-residue window width, the MTDIK matrix, and the 2.5 fold weighting of positions ‘*a*’ and ‘*d*’. For the PARCOIL server, a 28-residue window width and a *p*-score cut-off of 0.05 were used. Default settings were used for CCHMMPROF. The DrawCoil 1.0 server [58] was used to generate helical wheel representations of predicted coiled-coils.

### Analysis of sequence conservation

Two hundred distinct EIAV Rev amino acid sequences from the US, Ireland, and China, were retrieved from the NCBI GenBank protein database (see Additional file 2). A multiple sequence alignment of the central region of Rev was generated and a sequence logo corresponding to the coiled-coil motif (a.a. 82–109) was derived using the WebLogo server [59]. Sequence logos generated by WebLogo summarize the overall conservation of residues at each column position in a sequence alignment by depicting stacks of residues at each position: the height of each residue indicates its relative frequency. Relative frequencies are expressed in terms of information content, or bits, on the y-axis.

### Prediction of protein secondary structures

Secondary structure predictions for Rev proteins were obtained from the PSIPRED [70], ITASSER [32,34] and QUARK [36,37] webservers and manually aligned to generate a consensus secondary structure.

### Protein docking

The central region of Rev165 encompassing the predicted coiled-coiled motif (amino acids 82–109) was modeled with ITASSER. The ClusPro 2.0 docking server was used to generate dimeric structures, using default parameters [60-63].

### Expression and purification of EIAV Rev

MBP-Rev fusion proteins were cloned and expressed in *E. coli* strain Rosetta Gami in NZY media as described previously [26]. Following expression, cells were pelleted and resuspended in lysis buffer containing 25 mM HEPES pH 7.5, 200 mM NaCl, 2 mM beta-mercaptoethanol (BME), supplemented with 2 mM phenylmethylsulfonyl fluoride (PMSF) and Roche cOmplete® protease inhibitor cocktail tablet, according to the manufacturer’s protocol. The suspension was incubated with 1 mg/ml lysozyme on ice for 20 min and subjected to 10 cycles of freeze-thaw and 20 cycles of sonication. The suspension was clarified by centrifugation and mixed by rocking with Ni-NTA beads equilibrated in 50 mM Tris pH 8.0, 2 M NaCl, 2 mM BME, 0.1% Tween-20, 10 mM imidazole. After overnight incubation at 4°C, resin was rinsed with 5 sample volumes of equilibration buffer, washed with 5 sample volumes of wash buffer (50 mM Tris pH 8.0, 250 mM NaCl, 2 mM BME, 10 mM imidazole), and MBP-Rev fusion proteins were eluted in 50 mM Tris pH 8.0, 250 mM NaCl, 2 mM BME, 250 mM imidazole. Eluted protein samples were dialyzed against 50 mM Tris pH 8.0, 200 mM NaCl, 2 mM BME, 10% glycerol. The purity of all proteins preparations was confirmed by SDS-PAGE analysis.

### Blue native PAGE assay

Purified MBP-Rev protein samples were added to 6X Blue native sample loading buffer (12 mM EDTA, 120 mM NaCl, 120 mM Bis-Tris pH 7.0, 60% glycerol, and 0.5% Coomassie brilliant blue G-250 manufactured by Thermo Scientific, Waltham, MA) supplemented with 0.2% SDS. Samples were analyzed by electrophoresis in 8% Blue native polyacrylamide gels with 50 mM Bis-Tris pH 7.0 anode buffer and 50 mM Tricine, 15 mM Bis-Tris pH 7.0, 0.002% Coomassie brilliant blue G-250 cathode buffer.

### RNA binding assays

UV-crosslinking RNA binding assays were described previously [26]. Briefly, 2–4 μg purified MBP-Rev was incubated with 10^4^ cpm of ^32^P-labeled EIAV RRE RNA in binding buffer (10 mM HEPES-KOH, pH 7.5, 100 mM KCL, 1 mM MgCl_2_, 0.5 mM EDTA, 1 mM dithiothreitol, 50 μg/ml *E. coli* tRNA and 10% glycerol) for 20 min at room temperature. Following incubation, samples were UV-irradiated with 3×10^5^ μJ at 254 nm for 7 min, followed by treatment with 0.1 mg/ml RNase A at 37°C for 2 min. Samples were boiled in an equal volume of SDS for 5 min and separated in 12% SDS-PAGE in Tris-glycine buffer. Gels were fixed in 50% methanol-10% acetic acid, dried, and exposed to phosphorimager screens overnight. UV cross-linked complexes were detected using a PersonalFX scanner and Quality One software (Bio-Rad, Hercules, CA).

## Additional files

Additional file 1:
**Quality Assessment of Rev Models.** Complete list of elongated and globular models for all four sequences modeled (Revs 165, 135, ∆HVR, and FDD). The server from which each model was generated is listed. Each model’s quality scores, calculated using QMEAN and ProQ2, and the average of the two quality scores are also listed. The last column lists the calculated distance between ARM-1 and ARM-2. ^a^E: elongated; G: globular. ^b^: Servers used for protein prediction included QUARK [36,37]; ITASSER [32,34]; LOMETS [45]; and PROTINFO [46]. ^c^The QMEAN model quality assessment server is described in [47,48]. ^d^The ProQ2 model quality assessment server is described in [49]. ^e^Distance (in Ångstrom) was calculated for the pair of closest atoms between ARM-1 and ARM-2. Additional file 2:
**GenBank Accession Codes and Sequences of the EIAV Rev central region.** Complete list of sequences and Genbank accession numbers for the EIAV Rev central region (residues 76–120, based on EIAV R1) used for generating WebLogo of the coiled-coil motif.
