# Supplementary material for: Computational modeling suggests dimerization of equine infectious anemia virus Rev is required for RNA binding
Source: Retrovirology. 2014 Dec 23;11:115. doi: 10.1186/s12977-014-0115-7 (PMC4299382; doi:10.1186/s12977-014-0115-7)
Supplement: Additional file 2: — GenBank Accession Codes and Sequences of the EIAV Rev central region. Complete list of sequences and Genbank accession numbers for the EIAV Rev central region (residues 76–120, based on EIAV R1) used for generating WebLogo of the coiled-coil motif. [file 12977_2014_115_MOESM2_ESM.pdf]

GenBank ID	Sequence (aa 76-120)
AAG53100 (R1)	RRDRWIREQILQAEVLQERLEWRIRGVQQVAKEL
AAP34939	RRDRWIRGQILQAEVLQERLEWRIRGVQQAAKEL
AAP34940	RRDRWIRGQILQAEVLQERLEWRIRGVQQAAKEL
AAP34941	RRDRWIRGQILQAEVLQERLEWRIRGVQQAAKEL
AAP34942	RRDRWIRGQILQAEVLQERLEWRIRGVQQAAKEL
AAP34943	RRDRWIRGQILQAEVLQERLEWRIRGVQQAAKEL
AAP34944	RRDRWIRGQILQAEVLQERLEWRIRGVQQAAKEL
AAP34945	GRDRWIRGQILxQAEVLQERLEWRIRGVQQAAKEL
AAP34946	RRDRWIRGQILQAEVLQERLEWRIRGVQQVAKEL
AAP34947	RRDRWIRGQILQAEVLQERLGWRIRGVQQAAKEL
AAP34948	RRDRWIRGQILQAEVLQERLEWRIRGVQQAAKEL
AAP34949	RRDRWIRGQILQAEILQERLEWRIRGVQQAAKEL
AAP34950	RRDRWIRGQILQAEVLQERLEWRIRGVQQAAKEL
AAP34951	RRDRWIRGQILQAEVLQERLEWRIRGVQQAAKEL
AAP34952	RRDRWIRGQILQAEILQERLEWRIRGVQQAAKEL
AAP34953	RRDRWIRGQILQAEVLQERLEWRIRGVQQAAKEL
AAP34954	RRDRWIRGQILQAEVLQERLEWRIRGVQQAAKEL
AAP34955	RRDRWIRGQILQAEVLQERLEWRIRGVQQAAKEL
AAP34956	RRDRWIRGQILQAEVLQERLEWRIRGVQQAAKEL
AAB59743	RRDRWIREQILQAEVLQERLEWRIRGVQQVAKEL
ADK35796	RRDSWIRGQVQHAEVLQEQLKWRIRGVQQTAKEL
ADK35802	RRDSWIRGQVQHAEVLQEQLKWRIRGVQQTAKEL
ADK35819	RRDSWIRGQVQHAEVLQEQLKWRIRGVQQTAKEL
ADK35843	RRDSWIRGQVQHAEVLQEQLKWRIRGVQQTAKEL
ADK35849	RRDSWLRGQVQHAEALQEQLEWRIRGVQQTTKEL

ADK35855	RRDSWLRGQVQHAEALQEQLEWRIRGVQQTAKEL
ADK35861	RRDSWLRGQVQHAEALQEQLEWRIREVQQTAKEL
ADK35867	RRDSWLRGQVQHAEALQEQLEWRIRGVQQTAKEL
AAG53156	RRDRWIREQILQAEVLQERLEWRIRGVQQVAKEL
AAG53164	RRDRWIREQILQAEVLQERLEWRIRGVQQVAKEL
AAG53166	RRDRWIREQILQAEVLQERLEWRIRGVQQVAKEL
AAG53168	RRDRWIREQILQAEVLQERLEWRIRGVQQVAKEL
AAG53170	RRDRWIREQILQAEVLQERLEWRIRGVQQVAEEL
AAG53172	RRDRWIREQILQAEVLQERLEWRIRGVQQVAKEL
AAG53174	RRDRWIREQILQAEVLQERLEWRIRGVQQVAKGL
AAG53186	RRDRWIREQILQAEVLQERLEWRIKGVQQVAKEL
AAG53192	RRDRWIREQILQAEVLQERLEWRIRGVQQVAKEL
AAG53194	RRDRWIRGQILQAEVLQERLEWRIRGVQQVAKEL
AAG53196	RRDRWIREQILQAEVLQERLEWRIRGVQQVAKEL
AAG53198	RRDRWIREQILQAEVLQERLEWRIRGVQQVAKEL
AAG53200	RRDRWIRKQILQAEVLQERLEWRIRGVQQVAKEL
AAG53202	RRDRWIREQILQAEVLQERLEWRIRGVQQVAKEL
AAG53204	RRDRWIREQILQAEVLQERLEWRIRGVQQVAKEL
AAG53206	RRDRWIREQILQAEVLQERLEWRIRGVQQVAKEL
AAG53208	RRDRWIREQILQAEVLQERLEWRIRGVQQVAKEL
AAG53210	RRDRWIREQILQAEVLQERLEWRIRGVQQVAKEL
AAG53212	RRDRWIGEQILQAEVLQERLEWRIRGVQQVAKEL
AAG53214	RRDRWIREQILQAEVLQERLEWRIRGVQQVAKEL
AAG53216	RRDRWIREQILQAEVLQERLEWRIRGVQQVAKEL
AAG53218	RRDRWIREQILQAEVLQERLEWRIRGVQQVAKEL
AAG53220	RRDRWIREQILQAEVLQERLEWRIRGVQQVAKEL
AAG53228	RRDRWIREQILQAEVLQERLEWRIRGVQQVAKEL

AAG53230	RRDRWIREQILQAEVLQERLEWRIKGVQQVAKEL
AAG53232	RRDRWIREQILQAEVLQERLEWRIRGVQQVAKEL
AAG53234	RRDRWIREQILQAEVLQERLEWRIRGVQQVAKEL
AAG53236	RRDRWIREQILQAEVLQERLEWRIRGVQQVAKEL
AAG53238	RRDRWIREQILQAEVLQERLEWRIRGVQQVAKEL
AAG53240	RRDRWIREQILQAEVLQERLEWRIRGVQQVAKEL
AAG53242	RRDRWTREQILQAEVLQERLEWRIRGVQQVAKEL
AAG53244	RRDRWIREQILQAEVLQERLEWRIRGVQQVAKEL
AAG53246	RRDRWIREQILQAEVLQERLEWRIRGVQQVAKEL
AAG53248	RRDRWIREQILQAEVLQERLEWRIRGVQQVAKEL
AAG53250	RRDRWIREQILQAEVLQERLEWRIRGVQQVAKEL
AAG53252	RRDRWIREQILQAEVLQERLEWRIRGVQQVAKEL
AAG53254	RRDRWIREQILRAEVLQERLEWRIRGVQQVAKEL
AAG53256	GRDRWIREQILQAEVLQERLEWRIRGVQQVAKEL
AAG53258	RRDRWIREQILQAEVLQERLEWRIRGVQQVAKEL
AAG53260	RRDRWIREQILQAEVLQERLEWRIRGVQQVAKEL
AAG53262	RRDRWIREQILQAEVLQERLEWRIRGVQQVAKEL
AAG53264	RRDRWIREQILQAEVLQERLEWRIRGVQQVAKEL
AAG53266	RRDRWIREQVLQAEVLQERLEWRIRGVQQVAKEL
AAG53278	RRDRWIREQILQAEVLQERLEWRIRGVQQVAKEL
AAG53280	RRDRWIREQILQAEVLQERLEWRIRGVQQVAKEL
AAG53282	RRDRWIREQILQAEVLQERLEWRIRGVQQAAKEL
AAG53284	RRDRWIREQILQAEVLQERLEWRIRGVQQAAKEL
AAG53286	RRDRWIREQILQAEVLQERLEWRIRGVQQVAKEL
AAG53288	RRDRWIREQILQAEVLQERLEWRIRGVQQVAKEL
AAG53290	RRDRWIREQILQAEVLQERLEWRIRGVQQVAKEL
AAG53292	RRDRWIREQVLQAEVLQERLEWRIRGVQQVAKEL

AAG53294	RRDRWIREQVLQAEVLQERLEWRIRGVQQVAKEL
AAG53296	RRDRWIREQILQAEVLQERLEWRVRGVQQVAKEL
AAG53300	RRDRWIREQILQAEVLQERLEWRIRGVQQVAKEL
AAG53302	RRDRWIREQILQAEVLQERLEWRIRGVQQVAKEL
AAG53304	RRDRWIREQILQAEVLQERLEWRIRGVQQVAKEL
AAG53306	RRDRWIREQILQAEVLQERLEWRIRGVQQVAKEL
AAG53310	RRDRWIREQILQAEVLQERLEWRIRGVQQVAKEL
AAG53312	RRDRWIREQILQAEVLQERLEWRIRGVQQAAKEL
AAG53320	RRDRWIREQILQAEVLQERLEWRIRGVQQVAKEL
AAG53322	RRDRWIREQILQAEVLQERLEWRIRGVQQVAKEL
AAG53324	RRDRWIREQILQAEVLQERLEWRIRGVQQVAKEL
AAG53326	RRDRWIREQILQAEVLQERLEWRIRGVQQVAKEL
AAG53328	RRDRWIREQILQAEVLQERLEWRIRGVQQVAKEL
AAG53330	RRDRWIREQILQAEVLQERLEWRIRGVQQVAKEL
AAG53334	RRDRWIREQILQAEVLQERLEWRIRGVQQAAKEL
AAG53336	RRDRWIRGQILQAEVLQERLEWRIREVQQVAKEL
AAG53338	RRDRWIRGQILQAEVLQERLEWRIRGVQQAAKEL
AAG53340	RRDRWIREQILQAEVLQERLEWRIRGVQQVAKEL
AAG53342	RRDRWIREQILQAEVLQERLEWRIRGVQQVAKEL
AAG53344	RRDRWIREQILQAEVLQERLEWRIRGVQQVAKEL
AAG53346	RRDRWIRGQILQAEVLQERLEWRIRGVQQVAKEL
AAG53348	RRDRWIRGQILQAEVLQERLEWRIRGVQQVAKEL
AAG53350	RRDRWIREQILQAEVLQERLEWRIKGVQQVAKEL
AAG53352	RRDRWIREQILQAEVLQERLEWRIRGVQQVAKEL
AAG53354	RRDRWIREQILQAEVLQERLEWRIRGVQQVAKEL
AAG53356	RRDRWIREQILQAEVLQERLEWRVRGVQQVAKEL
AAG53358	RRDRWIREQILQAEVLQERLEWRIRGVQQVAKEL

AAG53376	RRDRWIREQILQAEVLQERLEWRIRGVQQAAKEL
AAG53378	RRDRWIREQILQAEVLQERLEWRVRGVQQAAKEL
AAG53380	RRDRWIREQILRAEVLQERLEWRIRGVQQAAKEL
AAG53384	RRDRWIRGQILQAEVLQERLEWRIRGVQQVAKEL
AAG53386	RRDRWIREQILQAEVLQERLEWRIRGVQQAAKEL
AAG53388	RRDRWIRGQILQAEVLQERLEWRIRGVQQAAKEL
AAG53390	RRDRWIREQILQAEVLQERLEWRIRGVQQAAKEL
AAA43006	RRDRWIRGQILQTEVLQERLEWRIRGVQQAAKEL
AFW99168	KRERWLRGQIQQAESLQEQLEWRIRGVQQSAEAL
AFW99174	KRERWLRGKIQQAESLQEQLEWRIRGVQQSAEAL
AFW99180	KRERWLRGQIQQAESLQEQLEWRIRGVQQSAEAL
AFW99186	KRERWLRGQIQQAESLQEQLEWRIRGVQQSAEAL
AAF28732	RRDRWIRGQILQAEVLQERLEWRIRGVQQVAKEL
AAC03765	RRDRWIRGQILQAEVLQERLEWRIRGVQQAAKEL
AAC24019	RRDRWIREQILQAEVLQERLEWRIRGVQQVAKEL
AAC24025	RRDRWIRGQILQAEVLQERLEWRIRGVQQVAKEL
AAF28723	RRDRWIRGQILQAEVLQERLEWRIRGVQQVAKEL
AAF28725	RRDRWIRGQILQAEVLQERLEWRIRGVQQAAKEL
AAF28726	RRDRWIRGQILQAEVLQERLEWRIRGVQQVAKEL
AAF28728	RRDRWIRGQAEVLQERLEWRIRGVQQVAKEL
AAF28729	RRDRWIRGQILQAEVLQERLEWRIRGVQQVAKEL
AAF28731	RRDRWIRGQILQAEVLQERLEWRIRGVQQVAKEL
AFW99519	KRERWLRGQIQQAESLQEQLEWRIRGVQQSAEAL
AFW99517	KRERWLRGQIQQAESLQEQLEWRIRGVQQSAEAL
AFW99515	KRERWLRGQIQQAESLQEQLEWRIRGVQQSAKAL
AFW99513	KRERWLRGQIQQAESLQEQLEWRIRGVQQSAEAL
AFW99511	KRERWLRGQIQQAESLQEQLEWRIRGVQQSAEAL

AFW99509	RRERWLRGKIQQAESLQEQLEWRIRGVQQSAKAL
AFW99507	KRERWLRGKIQQAESLQEQLEWRIRGVQQSAEAL
AFW99505	KRERWLRGQIQLAESLQEQLEWRIRGVQQSAEAL
AFW99503	RRERWLRGQIQQAESLQEQLEWRIRGVQQSAEAL
AFW99501	KRERWLRGQIQQAESLQEQLEWRIRGVQQSAKAL
AFV61765	KRDRWLRGRIQHAEQLQEQLEWRLKGVRQTAEAL
AAP35004	RRDRWIRGQILRAEVLQERLEWRIRGVQQAAKEL
AAP34999	RRDRWIRGQILRAEVLQERLDWRIRGVQQAAKEL
AAP34996	RRDRWIRGQILQAEVLQERLEWRIRGVQQAAKEL
AAP34995	RRDRWIRGQILQAEVLQERLGWRIRGVQQAAKEL
AAP34994	RRDRWIRGQILQAEVLQERLGWRIRGVQQAAKEL
AAP34993	RRDRWIRGQILQAEVLQERLGWRIRGVQQAAKEL
AAP34992	RRDRWIRGQILQAEVLQERLGWRIRGVQQAAKEL
AAP34981	RRDRWIRGQILQAEVLQERLEWRIRGVQQAAKEL
AAP34980	RRDRWIRGQILQAEVLQERLEWRIRGVQQAAKEL
AAP34979	RRDRWIRGQILQAEVLQERLEWRIRGVQQAAKEL
AAP34978	RRDRWIRGQILQAEVLQERLEWRIRGVQRTAKEL
AAM77610	RRDRWIRGQILQAEVLQERLEWRIRGVQQAAKEL
AAO14838	RRDRWIREQILQAEVLQERLEWRIRGVQQVAKEL
AAO14773	RRDRWIREQILQAEVLQERLEWRIRGVQQAAKEL
AAO14860	RRDRWIREQILQAEVLQERLEWRIRGVQQAAKEL
AAO14858	RRDRWIRERILQAEVLQERLEWRIRGVQQAAKEL
AAO14856	RRDRWIREQILQAEVLQERLEWRIRGVQQAAKEL
AAO14854	RRDRWIREQILQTEVLQERLEWRIRGVQQAAKEL
AAO14852	RRDRWIREQILQAEVLQERLEWRIRGVQQVAKEL
AAO14844	RRDRWIREQILQAEVLQERLEWRIRGVQQVAKEL
AAO14842	RRDRWIREQILQAEVLQERLEWRIRGVQQAAKEL

AAO14840	RRDRWIREQILQAEVLQERLEWRIRGVQQVAKEL
AAO14836	RRDRWIREQILQAEVLQERLEWRIRGVQQAVKEL
AAO14830	RRDRWIREQILQAEVLQERLEWRIRGVQQAAKEL
AAO14828	RRDRWIREQILQAEGLQERLEWRIRGVQQAAKEL
AAO14818	RRDRWIREQVLQAEVLQERLEWRIRGVQQAAKEL
AAO14817	RRDRWIREQILQAEVLQERLEWRIRGVQQVAKEL
AAO14815	RRDRWIREQILQTEILQERLEWRIRGVQQAAKEL
AAO14813	RRDRWIREQILQAEVLQERLEWKIRGVQQAAKEL
AAO14802	RRDRWIREQILQAEVLQERLEWRIRGVQQAAKEL
AAO14796	RRDRWIREQILQAEVLQERLEWRIRGVQQAAKKL
AAO14794	RRDRWIREQILQAEVLQERLEWRIRGVQQAAKEL
AAO14792	RRDRWIREQILQTEVLQERLEWRIRGVQQAAKEL
AAO14777	RRDRWIREQILQAEVLQERLEWRIRGVQQAAKEL
AAO14771	RRDRWIREQILQAEVLQERLEWRIRGVQQAAKEL
AAO14767	RHDRWIREQILQAEVLQERLEWRIRGVQQAAKEL
AAO14765	RRDRWIREQILQAEVLQERLEWRIRGVQQVAKEL
AAO14764	RRDRWIREQILQAEVLQERLEWRIRGVQQVAKEL
AAO14762	RRDRWIREQILQAEVLQERLEWRIRGVQQAAKEL
AAO14758	RRDRWIREQILQAEVLQERLEWRIRGVQQVAKEL
AAO14756	RRDRWIREQILQAEVLQERLEWRIRGVQQVAKEL
ADU02723	RRDSWLRGQVQHAEALQEQLEWRIRGVQQTAKEL
ADU02711	RRDSWLRGQVQHAEALQEQLEWRIRGVQQTAKEL
ADU02705	RRDSWLRGQVQHAEALQEQLEWRIRGVQQTAKEL
ADU02699	RRDSWLRGQVQHAEALQEQLEWRIRGVQQTAKEL
ADU02692	RRDSWIRGQVQLAEALQEQLEWRIRGVQQTAKEL
ADU02686	RRDSWIRGQVQHAEALQEQLEWRIRGVQQTAKEL
ADU02680	RRDSWLRGQVQHAEALQEQLEWRIRGVQQTAKEL

ADU02674	RRDSWLRGQVQHAEALQEQLEWRIRGVQQTTKEL
ADU02668	RRDSWLRGQVQHAEALQEQLEWRIRGVQQTAKEL
ADU02662	RRDSWIRGQVQHAEALQEQLEWRIRGVQQTAKEL
ADU02656	RRDSWLRGQVQHAEALQEQLEWRIRGVQQTAKEL
ADU02650	RRDSWLRGQVQHAEALQEQLEWRIRGVQQTAKEL
ADU02644	RRDSWLRGQVQHAEAPQEQLEWRTRGVQQTAKEL
ADU02638	RRDSWLRGQVQHAEALQEQLEWRIRGVQQTAKEL
AAB02404	RRDRWIRGQILQTEVLQERLEWRIRGVQQAAKEL
AAG02705	RRDRWIRGQILQAEVLQERLEWRIRGVQQAAKEL
AAA43027	RRDRWIRGQILQAEVLQERLEWRIRGVQQAAKEL
AAK21116	RRDSWLRGQVQHAEALQEQLEWRIRGVQQTAKEL
AAK21110	RRDSWLRGQVQHAEALQEQLEWRIRGVQQTAKEL
BAB12114	RRDRWIREQLLQAEVLQERLEWRIRGVQQAAKEL
BAB12108	RRDRWIREQILQAEVLQERLEWRIRGVQQAAKEL
